# A novel central line securement vest reduces line trauma and improves quality of life in patients with intestinal failure

**DOI:** 10.1002/jpr3.70165

**Published:** 2026-04-20

**Authors:** Ryan E. St. Pierre‐Hetz, Kimberly S. Ackerman, Angelica M. Cercone, Jane Anne Yaworski, Clifton W. Callaway, Jeffrey A. Rudolph, Mioara D. Manole

**Affiliations:** ^1^ Department of Emergency Medicine, Division of Pediatric Emergency Medicine Columbia University Vagelos College of Physicians and Surgeons New York New York USA; ^2^ Division of Pediatric Emergency Medicine UPMC Children's Hospital of Pittsburgh Pittsburgh Pennsylvania USA; ^3^ University of Pittsburgh Pittsburgh Pennsylvania USA; ^4^ Safar Center for Resuscitation Research, University of Pittsburgh Pittsburgh Pennsylvania USA

**Keywords:** central venous catheter, children, pediatrics, short‐gut syndrome, total parenteral nutrition

## Abstract

**Objective:**

We sought to assess the impact of a novel central line securement vest on the rate of line complications (trauma, infections, and replacements), and measures of quality of life (QOL) in pediatric patients with intestinal failure.

**Methods:**

We enrolled patients at a single tertiary pediatric center. We randomized 23 intestinal failure patients to a vest (*n* = 12, vest group) or traditional securement methods (*n* = 11, control group). Eleven patients in the vest group and 10 patients in the control group completed the study. Rates of trauma, infections, and replacements were calculated for each participant 12 months prior to and 12 months following enrollment, or for as long as the line remained in place and the vest was used. QOL scores were collected throughout the study.

**Results:**

The vest group had lower rates of trauma during the period of vest use versus prior to its use. The rates of infections and replacements were similar during vest use and prior to use. The control group had similar rates of trauma, infections, and replacements before and during the study period. The QOL scores in domains of activity level, overall QOL, and self‐perception were higher in the vest versus control group.

**Conclusions:**

In this pilot study, the use of a central line securement vest was associated with lower rates of line trauma. Several QOL domains showed better scores after the use of the vest in patients randomized to the vest versus the control group. Larger studies are warranted to assess the central line securement vest.

**Trial Registration:**

ClinicalTrials.gov identifier: NCT04522778; https://clinicaltrials.gov/study/NCT04522778.

## Abstract (highlights)

**What is Known:**

- Long‐term central venous catheters (CVCs) require multidisciplinary care and present a significant burden for both patients and their families.
- Currently, there are no devices available to protect long‐term CVCs from trauma or dislodgement.

**What is New:**

- Patients with intestinal failure experienced a reduction in CVC trauma during the months of using the central line securement vest, compared to the period before its use.
- A novel central line securement vest improved quality of life scores for patients using the vest, compared to those securing the CVC with the recommended standards of care.

## 1 INTRODUCTION

Central venous catheters (CVCs) are cuffed catheters that are tunneled subcutaneously to reach the vasculature. They are secured to the skin with an adhesive dressing.^1^, ^2^, ^3^ The distal catheter is exposed to the environment and is prone to complications such as line trauma, line fracture, or line infections, which can lead to line failure.^1^

CVC complications pose a great burden on families. Studies have noted that overall CVC failure rates are between 20% and 33% and are associated with significant healthcare costs.^4^, ^5^, ^6^ Children are also at a 3–4 fold increased risk of central line‐associated bloodstream infections (CLASBI) in the month following a repair.^7^ Additionally, patients' activities and their general quality of life (QOL) are impacted tremendously. Families of patients with CVCs report lower QOL scores in all psychosocial domains when compared to healthy counterparts.^8^

A novel central line securement vest was designed to secure CVCs against dislodgements and has been commercially available at a small scale since 2012. Many parents of children using this vest expressed to providers that it allows their child to be more active. In a small cohort of patients who had acquired the vest, we reported that the rate of line traumas and line infections were lower after versus before its use.^9^

Our primary goal was to assess the rate of complications in patients with intestinal failure while using the central line securement vest versus prior to using the vest. Our secondary goal was to evaluate the effect of the vest versus current securement methods on the QOL scores.

## 2 METHODS

### 2.1 Ethics statement

The research protocol was reviewed and approved by the Institutional Review Board (IRB) at the University of Pittsburgh, ensuring compliance with ethical standards for human subjects research.

### 2.2 Design

We designed a pilot single‐center study. Patients were randomized to the central line securement vest, henceforth referred to as the “vest,” and to the traditional central line securement method, henceforth referred to as the “dressing.” In each group, we compared the rate of line complications in the months before versus after the start of the study. Our primary outcome was the rate of line complications: line traumas, infections, and replacements before versus after enrollment. Our secondary outcome was QOL scores in the vest versus dressing groups.

### 2.3 Participants

Patients were eligible for inclusion if they had intestinal failure, a cuffed and tunneled CVC, and were between the ages of 0–18 years. Exclusion criteria included patients who had used the vest before the start of the study and females with breast development.

### 2.4 Patient enrollment

Patients were recruited from our institution's intestinal failure clinic between November 2020 and July 2021. At our institution, we exclusively use Broviac catheters, and thus all patients enrolled in our study had Broviac catheters. Based on our patient population, we expected an enrollment of less than 50 subjects. To maximize similar age distribution, patients were block randomized in three groups: 0–12 months, 13 months to 3 years, and 4–18 years. A block randomization algorithm was built in RedCap, and the investigators were blinded to study group allocation. Patients were randomized to either the vest or the dressing group.

### 2.5 Description of the vest

**Figure 1:**
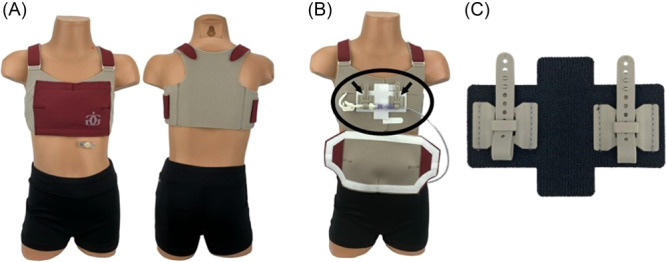
(A) Central line securement vest. (B) Circled is the dual safe strip. Arrows point to the dual safe strip, with the plastic securement locks. (C) Dual safe strip with the plastic securement locks.

The vest is the Gus Gear Central Line Vest® (Figure 1A,B). The vest has bilateral horizontal openings through which the CVC is placed (Figure 1B) to be secured inside the vest, and a dual safe strip that contains two plastic locks to secure the central line and the infusion line, respectively (Figure 1B,C). When closed, the vest encases the CVC in its entirety (Figure 1A). The vest is designed such that tension applied to the infusion line is absorbed by the locks and not transmitted to the CVC.

### 2.6 Control and intervention groups

The current standard of care for CVC securement consists of a sterile adhesive dressing. Patients randomized to the control group were asked to continue to care for their CVC as they had been instructed by their clinical team. Patients randomized to the vest were also asked to continue to care for their CVC as they had been instructed by their clinical team, and in addition, were instructed to wear the vest. They were fitted for the vest at the time of enrollment. The caretaker and the patient first observed vest fitting as demonstrated by a member of the study team on a manikin, then practiced vest placement on a manikin, and afterwards the caretaker was supervised while they placed the vest on the patient. The patients randomized to the vest were provided two vests at no cost to the participant, and were instructed to always keep one vest on, including during sleep and while bathing.

### 2.7 Data collection for the primary outcome of line complications

The primary outcome was the occurrence of three major line complications: line trauma, infections, and replacements in two groups of patients: the vest and dressing group, respectively. We elected to use patients as their own controls and compare the rate of complications before and after study enrollment, as line complications are patient‐dependent, with some patients having few events and others having multiple line complications. We used the vest group to assess the effect of the vest on line complications. We used the dressing group to assess the effect of increased familiarity with the line over time on line complications. The post‐enrollment line complication data were collected at each clinic visit and were validated with medical record review. During each clinic visit, patients and families filled out surveys and listed all incidents of line complications since the previous visit. Subjects' charts were reviewed monthly over the study period, and all line‐related complications were recorded. The line complications were recorded for a maximum of 12 months after randomization, or until the CVC was discontinued, whichever came first.

The pre‐enrollment line complications data were collected from medical records. Each patient's chart was reviewed for the 12 months prior to enrollment, or for the duration of having a CVC if it was less than 12 months. All line‐related complications were recorded. Additionally, all clinic notes were reviewed to identify potential additional complications that might not have been recorded in the medical record, such as when patients seek care elsewhere.

The event rate for line trauma, infection, and replacement (events per month) was computed for each patient before and after enrollment by dividing the number of events to the number of months before and after enrollment, respectively. For patients who stopped using the vest prematurely, the month of last vest usage was determined, and the event rate was calculated for the duration of usage.

### 2.8 Data collection for QOL

The secondary outcome for our study was the QOL. Our QOL survey was adapted from a validated survey by Baxter et al., modified for pediatric patients by Tran et al.^10^, ^11^ (Supplement 1). Some questions were adjusted to specifically ask about the impact of the dressing or the vest. The survey consisted of 27 questions with numerical responses. Questions assessed various domains of QOL including activities of daily living, ability to be active, social stressors, body image, and stress/parental anxiety. The patients and families completed QOL surveys at baseline and follow‐up visits. Families completed the surveys on a secure tablet using RedCap. If children were old enough and developmentally able, they would complete the surveys independently or with aid from family members. Investigators were blinded to responses. The follow‐up surveys were administered 3–6 times throughout the year for each patient. For patients who stopped using the vest prematurely, only surveys that were completed while the vest was used were analyzed. For this outcome, we collected the data prospectively and compared the QOL between the two groups.

### 2.9 Statistical analysis

Statistical Package for Social Sciences (SPSS Version 28.0) was used for descriptive analysis of primary outcome data. A paired *t* test was used to compare complications before and after enrollment.

Analysis for the QOL data was performed in STATA 16.1. QOL data were recorded in RedCap using ordinal scales. We compared responses to individual questions between the dressing group and vest group using ordinal logistic regression, adjusting for clustering of responses within a participant. QOL data are presented as the ordinal odds ratio (OR) and 95% confidence intervals for a unit change in response level for each question.

## 3 RESULTS

### 3.1 Patient enrollment and demographics

We screened 37 patients as potentially eligible for the study. Of these, seven patients were already using the vest and were excluded, and seven patients did not consent to the study. The remaining 23 patients were consented and randomized as follows: 11 patients were randomized to the dressing, and 12 patients were randomized to the vest. One patient randomized to dressing moved out of the area covered by our hospital the month after enrollment and was lost to follow‐up, and one patient randomized to the vest reported not ever wearing the vest. Both patients were excluded from the study.

Of the 10 patients randomized to the dressing, eight patients were followed for 12 months after randomization, and two patients had their central lines removed prior to 12 months. Of the 11 patients randomized to the vest, six patients wore the vest for the entire study (four patients for 12 months and two patients until the line was removed). Five patients wore their vest for less than 12 months, with a range of 2–6 months (Table 1).

**Table 1:** Demographics for all study participants.

Age	Gender	Diagnosis	Group	Months pre‐randomization	Months post‐randomization
1 yo	Female	Intestinal perforation	Vest	9	12
10 yo	Female	Intestinal hypoperistalsis	Vest	12	12
3 yo	Female	Gastroschisis	Vest	12	12
6 yo	Female	Necrotizing enterocolitis	Vest	12	12
12 yo	Female	Necrotizing enterocolitis	Vest	12	5
6 mo	Male	Necrotizing enterocolitis	Vest	2	8
11 yo	Male	Intestinal atresia	Vest	12	2
10 yo	Male	Gastroschisis	Vest	12	4
3 yo	Female	Gastroschisis	Vest	12	4
2 yo	Female	Necrotizing enterocolitis	Vest	12	6
5 mo	Male	Malrotation	Vest	2	5
11 yo	Male	Malrotation	Dressing	12	12
7 mo	Female	Malrotation	Dressing	5	12
8 yo	Female	Volvulus	Dressing	12	12
9 yo	Male	MEDNIK Syndrome	Dressing	12	12
15 yo	Male	Gastroschisis	Dressing	12	12
3 yo	Female	Necrotizing enterocolitis	Dressing	12	12
5 mo	Female	Malrotation	Dressing	4	12
8 mo	Female	Necrotizing enterocolitis	Dressing	7	12
14 yo	Male	Congenital diaphragmatic hernia	Dressing	12	7
6 yo	Male	Gastroschisis	Dressing	12	4

Abbreviations: MEDNIK, Mental retardation, Enteropathy, Deafness, Neuropathy, Ichthyosis, and Keratoderma; mo, month‐old; yo, year‐old.

### 3.2 Line complications

Line complication rates for the vest and dressing groups are summarized in Table 2. The rate of line trauma in the vest group decreased while using the vest (*p* = 0.03). In the dressing group, the rate of line trauma was similar before and after enrollment (*p* = 0.1). The rate of line infections was similar before and after enrollment (*p* = 0.15 and *p* = 0.3, respectively). The rate of line replacements in the vest group was similar before and during wearing the vest (*p* = 0.06), as well as in the dressing group (*p* = 0.3).

**Table 2:** Line complication rates pre‐ and post‐enrollment for vest and dressing group.

	Rates pre	Rates post	*p* Value
Vest			
Line trauma	0.25	0.05	0.03
Line replacements	0.18	0.08	0.06
Line infections	0.18	0.08	0.15
Dressing			
Line trauma	0.11	0.05	0.19
Line replacements	0.06	0.08	0.33
Line infections	0.10	0.08	0.33

The rates of line complications in the 12 months before enrollment were similar in the two groups for line trauma, line infections, and line replacements (*p* = 0.19, *p* = 0.4, *p* = 0.2, respectively).

### 3.3 QOL

In total, 54 QOL surveys were completed by all study participants. Of 27 questions (Supplement 1), five questions showed difference between the vest and dressing group (Table 3). These questions were related to family activity level (OR) = 109, patient's activity level (OR = 22, OR = 21), overall QOL (OR = 8), and self‐perception (OR = 0.2). The remaining questions in our surveys were not different between the two groups.

**Table 3:** Quality of life questions that had significant differences between vest and dressing groups.

Question	Odds ratio	Standard error	*Z* Value	*p* Value	95% confidence interval
How has the vest or dressing affected your ability as a family to participate in daily activities?	109.1	246.9	2.07	0.038	1.30–9193.65
How has the vest or dressing affected your child's ability to be active?	21.9	30.6	2.20	0.028	1.40–340.43
How has the vest or dressing affected your child's ability to participate in typical activities with peers or siblings?	21.3	28.6	2.28	0.022	1.54–294.88
How has your child's quality of life been affected by the dressing or vest?	7.8	6.634	2.40	0.016	1.455–41.431
How has the presence of a central line affected your child's body image?	0.2	0.135	−2.47	0.014	0.066–0.733

## 4 DISCUSSION

We assessed the impact of a central line securement vest on CVC‐related complications. To our knowledge, this is the first prospective study evaluating the performance of a vest designed to protect CVCs and improve QOL in patients with CVCs. Our data suggest that patients using the vest experienced lower rates of line trauma during its use compared to the period before. Additionally, patients who wore the vest reported better QOL outcomes, particularly in areas related to activity and overall well‐being, when compared to the control group.

This vest was initially manufactured on a small scale locally, and several families at our institution purchased it over the last decade. In these patients, we previously retrospectively analyzed complication rates before and after using the vest and found a reduction in line‐related complications.^9^ However, we recognized that evaluating the vest in families who independently purchased it may have introduced selection bias, as these families might have been more committed to rigorous CVC care or had additional resources for optimal patient management. To address this, we designed the current study, where we randomized patients from our intestinal care clinic to either use the vest or receive standard care. We chose to use a within‐patient control design, as complication rates are influenced by multiple patient‐specific factors.

We projected enrollment of a small number of patients in our single‐center study, and we anticipated that we could not control for various confounders. The patients were block randomized by age to control for age‐related differences in line complications. We used the dressing group as a control group because we felt it was necessary to assess whether increased familiarity with the line over time could influence outcomes. Specifically, patients and families might become more comfortable manipulating the line, which may reduce line complications over time. In the current study, we did not observe a decrease in line trauma over time in the control group. Therefore, our data suggest that the reduced rate of line trauma seen in the vest group is not solely attributable to increased familiarity with line care over time.

The vest was designed to protect the entire CVC from potential points of trauma, extending from the skin exit site to the hub of the CVC. The unique securement tabs (Figure 1) were engineered to achieve this goal using a plastic locking mechanism that relieves tension on the CVC. The vest secures the catheter by a series of fastening mechanisms, and the entire CVC is covered. Importantly, in our study, vest use was not associated with an increased rate of trauma, infection, or dislodgements. No patient reported unintentional dislodgement or trauma resulting from donning, doffing, or wearing the vest.

Patients who wore the vest experienced lower rates of line trauma requiring repair during the period of vest use, which was not observed in the control group. Rates of line infections and replacements were similar before and during the study for both vest and control groups.

While CVC‐related complications are a primary focus for preventative practices and comprehensive care, QOL for patients and families is another important outcome measure. Patients and families who require home total parenteral nutrition report lower QOL compared to healthy counterparts^3^ especially in the physical, emotional, and social domains.^12^ Our analysis of QOL outcomes revealed higher scores for patients wearing the vest versus controls, particularly in physical QOL, with improvements in the ability to engage in activities both independently and with family and peers. Parents also reported higher overall QOL scores for their children in the vest group compared to controls. Additionally, our data show that patients in the vest group reported a positive impact on body image related to their CVC.

Improvements in scores related to a patient's ability to be active are particularly important. Patients requiring total parenteral nutrition typically spend between 10 and 14 h per treatment day receiving infusion to ensure adequate nutrition and hydration.^13^, ^14^ Since CVCs are connected to large infusion bags, it is common for families and clinicians to restrict movement and activity during infusions. As a result, patients with intestinal failure often experience impaired psychomotor skills and developmental delays.^12^ The vest use was associated with increased odds of families rating higher levels of activity for both their child and family, compared to those using a dressing alone.

We followed patients prospectively for 12 months, recording data at each clinic follow‐up. Moreover, 55% of patients wore the vest for the entire duration of the study, while 45% wore it for less than 12 months (ranging from 2 to 6 months). Two patients, aged 10 and 11, reported that the vest was uncomfortable. A 3‐year‐old patient's family felt that the vest caused excessive sweating, while the family of a 2‐year‐old found the vest complicated to use. Additionally, the family of a 5‐month‐old infant did not believe the vest was helpful. Given the small sample size of this study, it is unclear whether the vest requires design improvements or if the families and patients had well‐established care routines prior to the vest's availability and did not adapt well to the addition of another element to CVC care. This issue may be better understood in future studies enrolling patients immediately after CVC insertion.

Our pilot study has several limitations. First, we enrolled subjects at a single institution and were able to recruit only a relatively small number of patients. Larger prospective multi‐institutional studies are needed. Although our intention was to follow patients for 12 months after enrollment, five patients stopped using the vest before completing the full duration. However, the families of these patients provided timely updates on the duration of vest use, allowing us to include the data collected during the months the vest was worn. We did not use objective measures of vest compliance during the months subjects were reported as using the vests. Due to the small sample size, we chose not to conduct an intention‐to‐treat analysis, as this could have introduced additional bias. Our study was also not designed to compare complication rates between the two groups; a larger study with that can employ that design is needed. Lastly, due to the nature of the study, after group assignment, investigator blinding was no longer possible.

## 5 CONCLUSION

In conclusion, our data suggest that the use of this novel central line securement vest is associated with reduced line trauma and improved QOL during the period of use. Larger studies involving diverse populations are needed to validate these findings.

## CONFLICT OF INTEREST STATEMENT

The authors declare no conflict of interest.

## Supporting information

Supplementary Information
