# Supplementary material for: A novel central line securement vest reduces line trauma and improves quality of life in patients with intestinal failure
Source: JPGN Rep. 2026 Apr 20:10.1002/jpr3.70165. Online ahead of print. doi: 10.1002/jpr3.70165 (PMC13398447; doi:10.1002/jpr3.70165)
Supplement: Supplementary file 1 — Supplementary Information [file JPR3-9999-0-s001.pdf]

Quality of Life Survey

Study ID Number: ____________________________ Patient Age: __________ Date: _____________

The central line securement device will be referred to as "vest" for the following questions.

Please check a box for each of the questions below:

During the past **Month(s)**:

	(1) Much worse	(2) Worse	(3) No change	(4) Better	(5) Much better
1. How has the vest affected your ability as a family to participate in daily activities?					
2. How has the vest affected your child's ability to be active?					
3. How has the vest affected your child's ability to participate in typical activities with peers or siblings?					
4. How has the vest affected your ability to keep your child's central line safe?					

During the past **Month(s)**:

	(1) Not at All	(2) A Little	(3) Quite a bit	(4) Very Much	(5) Not Applicable
1. Have you been worried about the safety of your child's central line?					
2. Has your child been able to take a long walk or play?					
3. Has your child been able to take a short walk outside of the house?					
4. Are you worried about your child's current health?					
5. Has your child needed to stay in bed or a chair during the day?					
6. Has your child needed help with eating, dressing, washing or using the toilet?					
7. Is your child able to socialize?					
8. Is your child able to do sports/exercise?					
9. Is your child able to attend school/pre-school?					
10. Does having a central line affect his/her participation in school?					
11. Does having a central line affect his or her learning at school?					
12. Is your child able to take part in hobbies or leisure activities?					
13. Is your child able to feel independent?					
14. Has the vest been easy to use?					
15. Has the vest been comfortable to wear?					

16. Has the vest kept you child’s central line safe?					
17.. Has the vest prevented your child from tampering with or touching the central line?					
18.. Has the vest enabled your child to be more active?					
19. . Has the presence of a central line affected their body image?					

Answer each of the following questions by either circling the relevant number or marking the relevant box:

During the past **<u>Month(s):</u>**

1. How would you rate your child’s quality of life on a scale of 1 to 10?

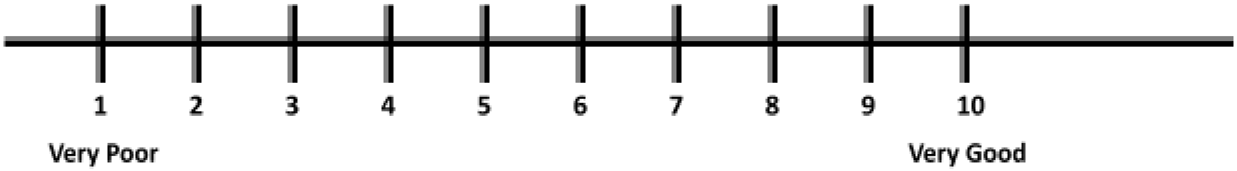

2. How would you rate your child’s ability to cope with day to day activities?

-5	-4	-3	-2	1	0	1	2	3	4	5
Very Poorly										Very Well

3. How has your child’s quality-of-life been affected by vest?

-5	-4	-3	-2	1	0	1	2	3	4	5
Very Much Worse										Very Much for the better

4. How has your child’s quality-of-life been affected by their underlying illness?

-5	-4	-3	-2	1	0	1	2	3	4	5
Very Much Worse										Very Much for the better
